# Obituary: Ciro de Quadros

**DOI:** 10.11604/pamj.2014.18.102.4712

**Published:** 2014-05-29

**Authors:** Raoul Kamadjeu

**Affiliations:** 1 The Pan African Medical Journal

**Keywords:** Obiturary, Ciro de Quadros

## Obituary

It is with great sadness that we announce the passing of a true public health hero, Dr Ciro de Quadros. Dr de Quadros died on 28 May 2014 in Washington DC, at the age of 74 years, after a long battle with cancer. He played an instrumental role in the eradication of polio in the Americas and has been actively involved in global immunization efforts with the Sabin Institute since 2003. The passing of Dr de Quadros is a tremendous loss for the Polio Eradication Initiative.

